# Supplementary material for: Single cell atlas of kidney cancer endothelial cells reveals distinct expression profiles and phenotypes
Source: BJC Rep. 2024 Mar 14;2:23. doi: 10.1038/s44276-024-00047-9 (PMC11524058; doi:10.1038/s44276-024-00047-9)
Supplement: Supplementary file 1 — Supplemental Information [file 44276_2024_47_MOESM1_ESM.docx]

**Supplemental Information**

**Single cell atlas of kidney cancer endothelial cells reveals distinct expression profiles and phenotypes**

Yuexin Xu^1^

Chris P. Miller^1^

Jun Xue^2,3^

Ying Zheng^2,3^

Edus H. Warren^1,4,5^

Scott S. Tykodi^5,6*^

Shreeram Akilesh^4,7*^

**Supplementary Methods**

**Suppl. Fig. 1. Quality control plots of scRNA-seq data.**

**Suppl. Fig. 2. Selected pathway gene expression in TECs and NECs.**

**Suppl. Fig. 3. The expression MHCs in TECs and NECs.**

**Suppl. Fig. 4. *IGFBP3* and *IGFBP5* survival curves from TCGA data.**

**Suppl. Fig. 5. The expression of tyrosine kinase inhibitor targets in TECs and NECs.**

**Suppl. Fig. 6. The composition of adherent CD45^+^ leukocytes on ECs.**

**Table S1. Patient demographics and sample description**

**Table S2. Endothelial cell Garnett markers**

**Table S3. Endothelial cell DEGs**

**Table S4. Endothelial cell count in published RCC single-cell RNAseq datasets**

**Table S5. qPCR primers**

**Supplementary Methods**

**Tissue sample processing**

Single-cell suspensions were prepared from primary tissues using the gentleMACS^TM^ tissue dissociator and tumor dissociation kit (Miltenyi Biotec, Bergisch Gladbach, Germany) using the “soft tissue” setting. PBMC were processed using Lymphocyte Separation Medium (Corning Inc., Corning, NY) density gradient centrifugation or SepMate PBMC Isolation (StemCell Technologies, Vancouver, Canada).

**Quantitative reverse-transcriptase PCR**

RNA samples of passage two and three TEC and NEC cultures and the corresponding primary NAT and RCC tumor cells were reverse-transcribed into cDNA libraries using SuperScript™ III One-Step RT-PCR System (ThermoFisher Scientific). All the qPCR primers (IDT, Newark, NJ) have been validated in previous publications (Table S4). The qPCR reactions were established with 20ng cDNA with PowerUp™ SYBR™ Green Master Mix (2X) (ThermoFisher Scientific) at 20 μL volume. The reactions were amplified for 40 cycles on QuantStudio5 Real Time PCR Systems (ThermoFisher Scientific) using the following PCR program: 95°C (10 minutes), followed by 40 cycles of 95°C (15 seconds) and 60°C (1 minutes).

**EC and immune cell co-culture**

For co-culture experiments, autologous CD45^+^ leukocytes were sorted from the same set RCC tumor and NAT tissue single-cell suspension and stained for CellTracker™ Green CMFDA Dye (Thermo Fisher Scientific). At day 0, NECs and TECs were stained by CellTracker™ Red CMTPX Dye (Thermo Fisher Scientific) and seeded at 0.1 million per well in endothelial media mixed with CTL cell media at 1:1 ratio. The VEGF concentration was 10ng/mL. The plates were cultured in Incucyte S3. 24 hours after seeding, the corresponding autologous CD45^+^ cells were added to the culture using the same media cocktail. On day2, the floating cells were harvested, imaged the plate on green and red channel on incucyte. 16 separate images were taken on each of the wells. We then trypsinized the adherent cells, flow stained for immune cell population to analyze the adherent CD45^+^ cell composition using the following antibody panel each at 1:20 dilution at BD FACSymphony™ analyzer (BD Biosciences): PE-Cy7 mouse anti-human labeled CD19 (clone HIB19, BD Biosciences), BV605 labeled mouse anti-human CD25 (clone 2A3, BD Biosciences), PerCP-Cy5.5 labeled mouse anti-human CD4 (clone RPA-T4, BD Biosciences), BV786 labeled mouse anti-human CD8 (clone RPA-T8, BDBiosciences), APC-R700 labeled mouse anti-human CD56 (clone NCAM16.2, BD Biosciences) and AF647 labeled mouse anti-human CD68 (clone Y1/82A, BD Biosciences).

**Bulk RNA-sequencing**

Three pairs of TEC and NEC cultures at passage three as well as the corresponding primary normal kidney and RCC tumor tissues were trypsinized to single-cell suspensions. The primary ECs were enriched by sorting on the DAPI^-^CD31^+^CD144^+^ population. RNA samples were extracted using RNeasy Plus Mini kit (Qiagen, Hilden, Germany) to remove genomic DNA. 50ng RNA samples at 5ng/μL concentration were reverse-transcribed by poly-A selection using oligo dT primer. Clontech SMARTv4 (Takara Bio) and Nextera XT (llumina) kits are used to construct the library according to the manufacture’s protocol. The sample pool was sequenced at 30 million 50PE reads per sample on an Illumina Nextseq P2 flowcell.

**Supplemental Figures**

**Suppl. Fig. 1. Quality control plots of scRNA-seq data. (A)** Tissue origin of each cell. HUVEC cells were spiked into samples extracted from tumor and normal kidney (NAT). **(B)** HTO tag assignment of each cell. **(C)** Cell lineage marker expression of each cluster. **(D)** UMAP plots of all cells with barcodes colored by four patients, **(E)** two separate libraries, and **(F)** *ACTB* expression.

**Suppl. Fig. 2. Selected pathway gene expression in TECs and NECs.** The expression of **(A)** *MMP* and **(B)** *ADAMT* genes in TECs and NECs. **(C)** The expression of angiogenic pathway genes (GO:0001525) in TECs and NECs.

**Suppl. Fig 3. The MHC gene expression in TECs and NECs.** The expression of MHC genes on TECs and NECs in **(A)** single-cell RNAseq dataset, and **(B)** RNA-seq dataset with the expression level on cultured TECs and NECs.

**Suppl. Fig 4. *IGFBP3* and *IGFBP5* survival curves from TCGA data.** The expression of **(A)** *IGFBP3* and **(B)** *IGFBP5* in all cell clusters. **(C)** The expression of IGF pathway genes in individual cell types. The protein expression of **(D)** IGFBP3 and **(E)** IGFBP5 in normal human organs and tumors. Images are from the Human Protein Atlas (v22.0.proteinatlas.org). The overall survival curve stratified by **(F)** *IGFBP3* and **(G)** *IGFBP5* from the TCGA ccRCC dataset (KIRC). The cohort was divided into two equal size groups by the expression of the mRNA. The results here are based upon RNAseq data generated by the TCGA Research Network: <https://www.cancer.gov/tcga>.

**Suppl. Fig 5. The expression of tyrosine kinase inhibitor targets in TECs and NECs. (A)** Heatmap of tyrosine kinase inhibitor target expression in cultured TECs/NECs and primary TECs/NECs. Gene expression was calculated by the populational average and centered by Z-score.

**Suppl. Fig 6. The composition of adherent CD45^+^ leukocytes on ECs (A)** The flow gating strategy of different leukocytes populations. **(B)** The % of total cells of each adherent leukocyte populations on the TECs and NECs.
